# Stereotactic Gamma Ray Radiosurgery to the Centromedian and Parafascicular Complex of the Thalamus for Trigeminal Neuralgia and Other Complex Pain Syndromes

**DOI:** 10.7759/cureus.6421

**Published:** 2019-12-19

**Authors:** Eduardo E Lovo, Boheris Torres, Fidel Campos, Victor Caceros, William A Reyes, Kaory C Barahona, Claudia Cruz, Juan Arias, Eduardo Alho, William O Contreras

**Affiliations:** 1 Radiosurgery, International Cancer Center, Diagnostic Hospital, San Salvador, SLV; 2 Neurosurgery, International Cancer Center, Diagnostic Hospital, San Salvador, SLV; 3 Radiation Oncology, International Cancer Center, Diagnostic Hospital, San Salvador, SLV; 4 Anesthesia and Pain Management, Hospital De Diagnóstico, San Salvador, SLV; 5 Pain Management, International Cancer Center, San Salvador, SLV; 6 Functional Neurosurgery, University of Sao Paulo Medical School, Sao Paulo, BRA; 7 Functional Neurosurgery, Clinica Foscal Internacional, Bucaramanga, COL

**Keywords:** radiosurgery, functional radiosurgery, pain, trigeminal neuralgia, neurosurgical management, thalamotomy

## Abstract

Introduction

We report our initial series of patients treated with radiosurgery to the Centromedian (CM) and Parafascicular (Pfc) Complex (CM-Pf) of the contralateral thalamus mainly for trigeminal neuralgia that had failed most known forms of conventional treatments. The coordinates were co-registered to a three-dimensional atlas of the thalamus in order to have a better comprehension of isodose curves distribution.

Methods

A fully automated rotating gamma ray unit was used to deliver a high dose of radiation (140 Gy) using a 4-mm collimator to the CM-Pf of the contralateral thalamus in 14 patients suffering from refractory trigeminal pain and other complex pain syndromes. The best stereotactic coordinates were plotted in a thalamic three-dimensional atlas space along with isodose curves corresponding to 50% of the dose prescription and the dose gradient.

Results

From November 2016 to July 2019, 14 patients experiencing severe forms of different pain syndromes were treated, and 10 were eligible for follow-up evaluation. Pain deriving from trigeminal neuralgia was present in the majority (80%) of patients and from other complex pain syndromes in the rest (20%). Median follow-up was 384 days (range: 30-994). The Visual Analogue Scale (VAS) score before treatment was 9 (range: 7-10) and standardized to 10. Before treatment, all the patients had a Barrow Neurological Institute Pain Scale (BNI) of 5 (V). The median years suffering from pain was 4.5 years (range: 1-15), the number of procedures including radiosurgery to the trigeminal nerve before thalamotomy was four (range: 1-10). Most patients (90%) reported some form of relief, the average VAS at the time of response was 3.5 (range: 0-9), and the average time to response was 67.3 days (range: 2-210). The neuromodulation effect of radiation was seen in 60% of patients. The average BNI score at response was 2.7 (range: 1-5). The final VAS score at last follow-up was 5.5 (range: 0-10) in six patients. In four patients (40%), the procedure had failed with a final BNI of IV, and V, three patients (30%) had excellent response (BNI of I), and three patients (30%) had worthwhile results with BNI of IIIa and IIIb. The total success rate (BNI of I to IIIb) was 60%, and the number of patients experiencing more than 50% of pain reduction at final follow-up was five (50%). Excluding both patients that were treated for pain outside of trigeminal neuralgia, 75% of the patients responded. The best coordinates on average were X: 5.5 mm from the thalamic border, Y: 3.7 mm anterior to the posterior commissure, and Z: 3.7 mm from the intercomissural line. There were no complications to report.

Conclusion

Radiosurgery to the CM-Pf of the thalamus was demonstrated to be a safe and relatively effective alternative to treat refractory trigeminal neuralgia. Further studies are needed to optimize target dimensions based on the three-dimensional studies of isodose curves as well as coordinates. Longer follow-up is necessary to evaluate recurrence rates that could not be reached.

## Introduction

Trigeminal neuralgia can constitute a true challenge for all modalities of treatments; the best results emerging from microvascular decompression, radiosurgery or percutaneous treatments still leave behind a small number of patients who do not obtain relief, particularly those suffering from secondary trigeminal neuralgia [1-8]. Most of these patients have constant pain, are usually heavily medicated and have already been treated with multiple modalities. Some of these patients are experiencing anesthesia dolorosa that can follow iatrogenic damage to the peripheral or, less often, central portions of the nerve.

What is more popularly known as “Medial thalamotomy” using radiosurgery for the management of pain dates to the origins of Gamma Knife® radiosurgery in the hands of Leksell [9]. The fundamental basis was to create a lesion in the medial structures of the thalamus as they are considered relay nuclei in the interpretation of pain, especially the Centromedian (CM) and Parafascicular (Pfc) Complex (CM-Pf) [9-13]. Thalamotomy or stimulation of the medial structures of the thalamus for neurogenic pain has been performed using surgery, radiosurgery, and high-focused ultrasound for chronic deafferentation pain usually arising from neurological damage [9-16]. In the 1990s, Young et al. delivered high doses of radiation to different targets in the thalamus that included interlaminar, CM, and dorsomedial regions as they are considered areas of the relay of the paleospinothalamic tracts that carry sensitive and affective information regarding chronic pain [10,11]. The renewed interest in this region and the use of radiosurgery was spurred by a recent publication by Urgosik and Liscak in a relatively large series of patients mostly suffering from complex or refractory trigeminal pain [13]; they targeted the CM-Pf considered to be located 4 mm to 6 mm lateral to the wall of the third ventricle, 8 mm posterior to the midpoint, and 2 mm to 3 mm superior to the intercommissural line.

We describe our initial experience with radiosurgical medial thalamotomy mainly for refractory trigeminal neuralgia using a rotating gamma ray system and describe the isodose curves coverage in relation to the CM-Pf with regards to the best clinical results that were obtained.

## Materials and methods

Patient selection

We conducted a retrospective study regarding radiosurgery to the CM-Pf for patients referred by pain and palliative medicine suffering mainly from anesthesia dolorosa in the trigeminal region or refractory trigeminal neuralgia that had completely failed radiosurgery to usual targets within the intracranial posterior fossa segments of the nerve at habitual doses of radiation (90 Gy). A complete failure of radiosurgery was defined as no perceived beneficial effect (a Barrow Neurological Institute Pain Scale [BNI] of IV or V) six months after treatment. Other complex pain was included such as anesthesia dolorosa of the lesser occipital nerve and complex regional pain syndrome (CRPS) of the superior limb after failed cervical surgery. All patients were declared refractory to medical treatment including the addition of opioid therapy in whom no reasonable alternative intervention such as surgery was feasible. The trial was approved by the hospital’s ethical committee in November 2016. The inclusion criteria were patients with refractory trigeminal neuralgia with “complete failure” after radiosurgery to the nerve, patients that presented with anesthesia dolorosa secondary to surgical procedures, nerve sectioning or one case where there were no identifiable nerve portions due to tumor on stereotactic magnetic resonance imaging (MRI) studies. Also, the patients’ pain had to be categorized as severe (scoring 7 to 10) on the Visual Analog Scale (VAS) despite best medical algology practice and optimal pharmacological management. In addition, they had to be 18 years or older to enroll and needed to possess adequate family support to understand the expectations from treatment, the standardization of VAS to 10 before treatment, and how follow-up would be conducted. Interviews with patients and family members were routinely performed by the leading neurosurgeon and pain specialist. The cases were reviewed and approved by an ad-hoc committee that included a pain and palliative care specialist, neurosurgeon, and radiation therapy staff. Our main endpoints were the reduction of the VAS score by at least 50% and a reduction in pain medicine consumption as well as a monthly emergency room attendance of at least 25%.

Radiosurgical technique

**Figure 1:**
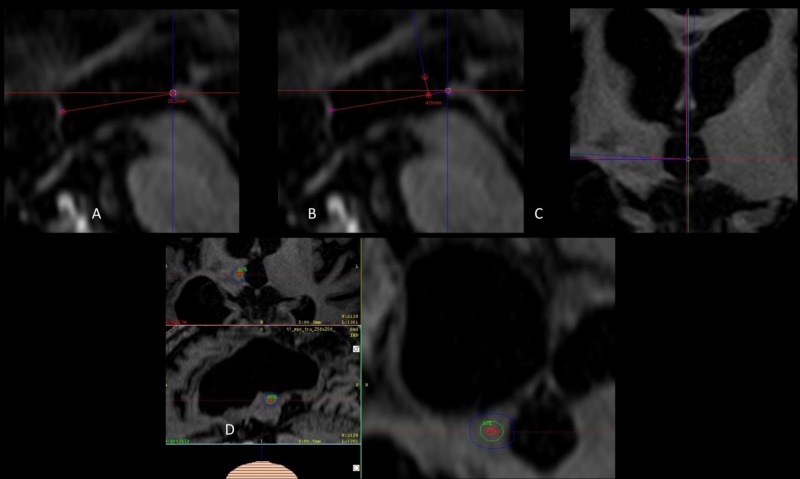
Coordinates and shoot placement (A) Sagittal view of the ICL. (B) Measurements of coordinates that correspond to Y and Z. (C) Axial view with correction of angulations to determine X from the border of the thalamus. (D) Three-dimensional view of the 4-mm shoot, the green isodose curve represents 50% of the dose (70 Gy), the most external blue isodose curve represents dose gradient to 25% of the dose (35 Gy). ICL: Intercomissural line

On the day of the procedure, patients fasted six hours and were prepared for sedation by the anesthesiologist. Under local anesthesia, an Infini™ stereotactic frame (Masep Medical Company, Shenzhen, China) was placed by a neurosurgeon, and the MR acquisition was performed with a 1.5-Tesla Avanto™ (Siemens Corporation, Erlangen, Germany). Normally only one volumetric T2 of a 6-mm slice thickness with no spacing of the head (apex to foramen magnum) was acquired for defining the skin; T1 and T2 constructive interference in steady state of 1-mm slice thickness with no spacing from the corpus callosum covering the thalamus below the trigeminal nerve were also acquired. Images were transferred to the treatment planning station (Superplan®). For planning, the anterior commissure and posterior commissure (PC) were identified in the axial plane, then the images were transformed into the sagittal plane using fusion tools and the intercomissural line (ICL) was drawn between T1 and T2 sequences. The distance from the PC was taken anteriorly along the ICL (usually 4 mm) and confirmed and adjusted by taking 8 mm posterior to the midpoint of the ICL; this was identified as Y. A 90° angle was traced from the PC to the Y along the ICL, and Z was determined 3 to 4 mm above the ICL. Finally, images were reoriented to axial views, and X coordinates were 4 to 6 mm lateral from the contralateral thalamic border to the side of the pain. Using the 4-mm collimator, a single shot was placed and adjusted based on anatomy similitudes to the initial cases that provided the best response. Due to safety mechanisms specific to the Superplan® and the use of the prescribed high dose, we needed to place two shots in the same coordinates. The plan was reviewed and approved by radiation oncology and neurosurgery. The prescribed dose of 140 Gy to Dmax was administered to all patients with a gamma angle regularly fixed at 90° (Figure 1).

After treatment, the frame was removed, and the patient was sent home with their usual pain medication.

Patient follow-up

Patients were contacted 72 hours and every month after treatment until response, recurrence, or the end of the initial follow-up period. Neuromodulation effect by radiation (NER) was defined as a prompt relief of at least 25% of pain, usually occurring immediately after treatment or in a brief time span of fewer than 30 days. NER was usually categorized by being transient as pain relief was short, and pain recurs until definite sustained pain relief was obtained or failure of treatment was documented.

Thalamic three-dimensional atlas plotting

**Figure 2:**
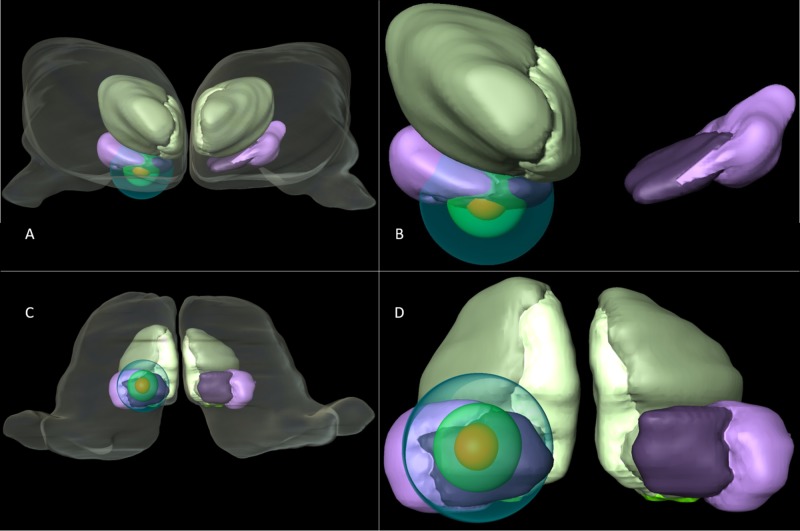
Three-dimensional reconstruction of the CM-Pf and isodose curves (A) The thalamus is seen from an anterior perspective of the centromedian nucleus in light purple, parafascicular complex in dark purple, the medial thalamic group in light green. The different isodose curves corresponding to 100 Gy, 70 Gy and 35 Gy from the most inner circle to the outmost. (B) Close-up of an anterior perspective of the area of interest with the isodose curves. (C) The thalamus in a ventral perspective is demonstrated with the nuclear subdivisions of the thalamus with interest only in the CM-Pf complex. (D) Close-up of the ventral perspective of the area of interest with the isodose curves. CM-Pf: Centromedian and Parafascicular

The thalamic reconstruction is part of the São Paulo-Würzburg Atlas of the Human Brain, details on the histological processing and image registration pipelines can be found elsewhere [14]. Briefly, a histologically processed brain was MRI scanned postmortem in situ and registered into a computational pipeline. To illustrate the structures affected by the thalamotomy, the average of the best coordinates of the isocenter was plotted into the histological atlas with the accompanying three-dimensional isodose curves with the most internal in red corresponding to 100 Gy, the green isodose line corresponding to 50% of the dose prescribed (or 70 Gy), and blue sphere corresponding to the dose gradient of 25% of the dose prescribed (or 35 Gy). It is possible to see that the hotspot is centered in the Pfc in dark purple, with the isodose curve corresponding to the dose gradient from 35 Gy to 70 Gy reaching the CM nucleus in light purple and the medial thalamic group in green tones (Figure 2).

## Results

From November 2016 to July 2019, 14 patients were treated, and 10 were eligible for follow-up evaluation, eight (80%) were female, and the mean age at treatment was 45 years (range: 22-72 years). The pain duration was 4.5 years (range: 1-15 years); the most affected side was the left side in seven patients (70%). Pain deriving from refractory or anesthesia dolorosa of the trigeminal neuralgia was present in the majority of the studied population (80%). One patient in the trigeminal pain group had anesthesia dolorosa secondary to a left maxillary sinus carcinoma resection and was the only patient categorized as a palliative oncological patient expected to survive less than six months. As for the other two patients eligible for follow-up who did not have trigeminal neuralgia pain, one experienced an anesthesia dolorosa of the left lesser occipital nerve do to multiple nerve blocks, radiofrequency, and subsequent surgical nerve sectioning and the other patient suffered from CRPS to the right superior limb after multiple cervical spine interventions and refusal to evaluate conventional neuromodulation therapy. The mean number of surgical or percutaneous procedures (either nerve blocks or in combination with radiofrequency and radiosurgery) to the trigeminal nerve was 4.6 (range: 1-10). The VAS score was 9 (range: 8-10) despite best medical treatment by pain and palliative medicine, and the VAS was standardized to 10. Before treatment, all the patients had a BNI of five (V). Of the patients suffering from trigeminal neuralgia, four experienced “complete failure” of radiosurgery to the trigeminal nerve at a habitual dose of 90 Gy. Median follow-up was 384 days (range: 30-994 days; Table 1).

**Table 1:** Patient characteristics BNI: Barrow Neurological Institute Pain Scale; CRPS: Complex regional pain syndrome; ER: Emergency room; NME: Neuromodulation effect; SRS: Stereotactic radiosurgery to nerve; Thalamo: thalamotomy; TN: Trigeminal neuralgia; VAS: Visual analog scale.

Patients	Pathology	Age	Years before Thalamo	VAS before Thalamo	BNI before Thalamo	Procedures before Thalamo	SRS before Thalamo	Refractory to meds	Days to response	NME	NME days	Final VAS	Final BNI	Follow up	Medicine reduction (at least one intake)	ER reduction (at least 25%)
1	TN post-surgery	22	2	10	V	4	Yes	Yes	91	No	-	5	IIIa	994	Yes	Yes
2	Refractory TN	48	6	10	V	3	Yes	Yes	28	No	-	6	IIIb	735	Yes	Yes
3	CRPS	46	3	10	V	3	No	Yes	28	Yes	15	10	V	371	Yes	Yes
4	TN post-surgery	56	10	10	V	1	No	Yes	36	Yes	15	7	IV	436	Yes	Yes
5	Anesthesia Dolorosa TN	32	1	10	V	1	No	Yes	2	Yes	2	0	I	30	Died	Died
6	Anesthesia Dolorosa TN-Hz	72	15	10	V	10	No	Yes	33	Yes	10	1	I	322	Yes	Yes
7	Anesthesia Dolorosa TN	41	8	10	V	6	No	Yes	33	Yes	7	10	V	384	Yes	No
8	Anesthesia Dolorosa Occipital Nerve	59	2	10	V	10	No	Yes	62	No	-	10	V	265	No	Yes
9	TN tumor compression	29	3	10	V	3	Yes	Yes	30	Yes	5	0	I	779	Yes	Yes
10	Refractory TN	46	7	10	V	5	Yes	Yes	65	No	-	2	IIIa	231	Yes	Yes

After treatment, almost all patients (90%) reported some form of relief. Six patients (60%) achieved the 50% or more pain relief threshold defined by the study characteristics as success. For four patients (40%), the procedure had failed as final BNI was IV and V, and pain never improved (VAS score less than 7). The NER was seen in 60% of the patients at a mean time of 8.5 days (range: 2-15 days) with a VAS score of 3 (range: 0-7). The duration of the NER effect lasted 15 days. In one patient, in whom thalamotomy was performed due to an inability to visualize the trigeminal nerve due to tumor, the pain had not recurred after 808 days. Another patient, in whom thalamotomy was used as a palliative alternative for anesthesia dolorosa secondary to a left maxillary sinus carcinoma resection, responded at 48 hours and died at 31 days after the procedure without pain.

The average VAS score at the time of sustained response was 3.5 (range: 0-9); at 67.3 days (range: 2-210 days), the BNI score was 2.7 (range: 1-5). The final VAS score at last follow-up was 5.5 (range: 0-10). Three patients (30%) had an excellent response (BNI I), and three patients (30%) had worthwhile results (BNI IIIa, IIIb). The total success rate by BNI I to IIIb was 60%, and the success rate by more than 50% of pain relief at last follow-up was 50%. One patient within the success group recurred after 798 days. Excluding both patients that were treated for pain different than trigeminal neuralgia, 75% of the patients responded. At last follow-up, one patient had died, and the rest had reduced their monthly visits to the emergency room at least 25% (range: 0%-100%), and 90% of the patients had a reduction of at least one of their pain medications, even in those with failed treatment failure (BNI IV and V).

The best coordinates, on average, were X: 5 mm from the thalamic border, Y: 3.7 mm anterior to the posterior commissure, and Z: 3.7 mm from the intercommisural line. There were no statistical differences in coordinates to those whose treatment was considered a failure (Table 2).

**Table 2:** Coordinates based on clinical results in trigeminal neuralgia Coordinates: X based on thalamic border, Y is distance anterior to the posterior commissure, and Z is positive to the inter-commissural line. Not TN: Not trigeminal neuralgia pain

Coordinates	X	Y	Z
Best results	5	4.2	2.6
	5.6	4.4	4.7
	5.1	3.9	2.9
	5.5	2.8	4.5
Good	4	3.4	3.8
Average	5	3.7	3.7
Failed	4.8	3.9	4.5
Not TN	4	3.1	3.1
Not TN	4.6	3.8	4.1

There were no complications to report.

## Discussion

Lesions and stimulation to the CM-Pf of the thalamus in order to alleviate intractable pain have a long neurosurgical tradition [9-13,15-17]. Radiosurgery has been used on different targets in the region, and doses have varied, but, to a lesser extent recently, it has been used to treat different forms of pain arising from oncological and non-oncological origins yielding relatively similar results regarding pain control (Table 3).

**Table 3:** Comparative radiosurgery series that have been published

Author	Patients	Pathology	Target	Dose	Initial result	Final results
Steiner et al. 1980 [9]	49	Malignant	Centromedian-parafascicular complex	140-250 Gy	36-67%	8-37%
Young et al. 1995 [11]	20	Chronic intractable pain	Centromedian-parafascicular complex and intralaminar	140-180 Gy		65%
Urgosik and Liscak 2018 [13]	30	Mainly trigeminal pain	Centromedian-parafascicular complex	145-150 Gy	(At least 50% pain reduction) 43%	30%
Current series	10	Mainly trigeminal pain	Centromedian-parafascicular complex	140 Gy	(At least 50% pain reduction) 70%	50%

This protocol was implemented in our center as an alternative for those patients for whom radiosurgery to the nerve completely failed. This number in our initial series hovered between 14% and 16% of the population [18]. Early results of this series encouraged further treatments in anesthesia dolorosa along the trigeminal nerve distribution secondary to surgery and, more recently, are being used sometimes in combination with the simultaneous trigeminal nerve irradiation for complex cases where the patient’s life might be in danger either from inanition due to pain or tumor invasion to the trigeminal path where surgery or percutaneous treatments are not an option. What has been known as “medial thalamotomy” (i.e., radiosurgery to the CM-Pf and the medial region) has been considered safe in the vast majority of patients that have been reported in the literature as no complications have been described with lesser doses (140-150 Gy) using a single 4-mm isocenter to the contralateral thalamus. In the present study, the three-dimensional plotting of the isodose curve makes us believe that a larger zone could be irradiated, possibly combining a “shoot within a shoot” of an 8-mm and 4-mm beam covering more of the CM with a higher dose. While the results might be considered mediocre, these patients had no other viable alternative, and success, although low, was registered where all else had failed.

Possible neuromodulation effects by radiation, with sub-necrotic doses of radiation have been proposed by several authors. Ill-defined and poorly understood clinical results in epilepsy and trigeminal neuralgia patients who report immediate relief of symptoms or pain at the end of the radiosurgical procedure have been witnessed by most who practice functional radiosurgery and have been reported by some authors [19-21]. Ohye attributed the rapid tremor improvement in some patients (days after radiosurgery to the ventral intermediate nucleus) to psychological effects, given the normal expected timeline is six months to generate a lesion by a high dose of radiation and note any clinical effect [22]. In this study, 60% of the patients presented a substantial improvement in pain a few days after treatment, which is too short of a time to attribute effect to radiation damage. Therefore, we consider this to be neuromodulation effect by radiation. These patients often reported improvement in pain, and, in most cases, the pain recurred until the definitive initial response ensued. Of the 14 patients we treated, we saw this effect in eight patients - even in those whose pain recurred at a brief time and never settled back down (considered a failure of the treatment).

**Figure 3:**
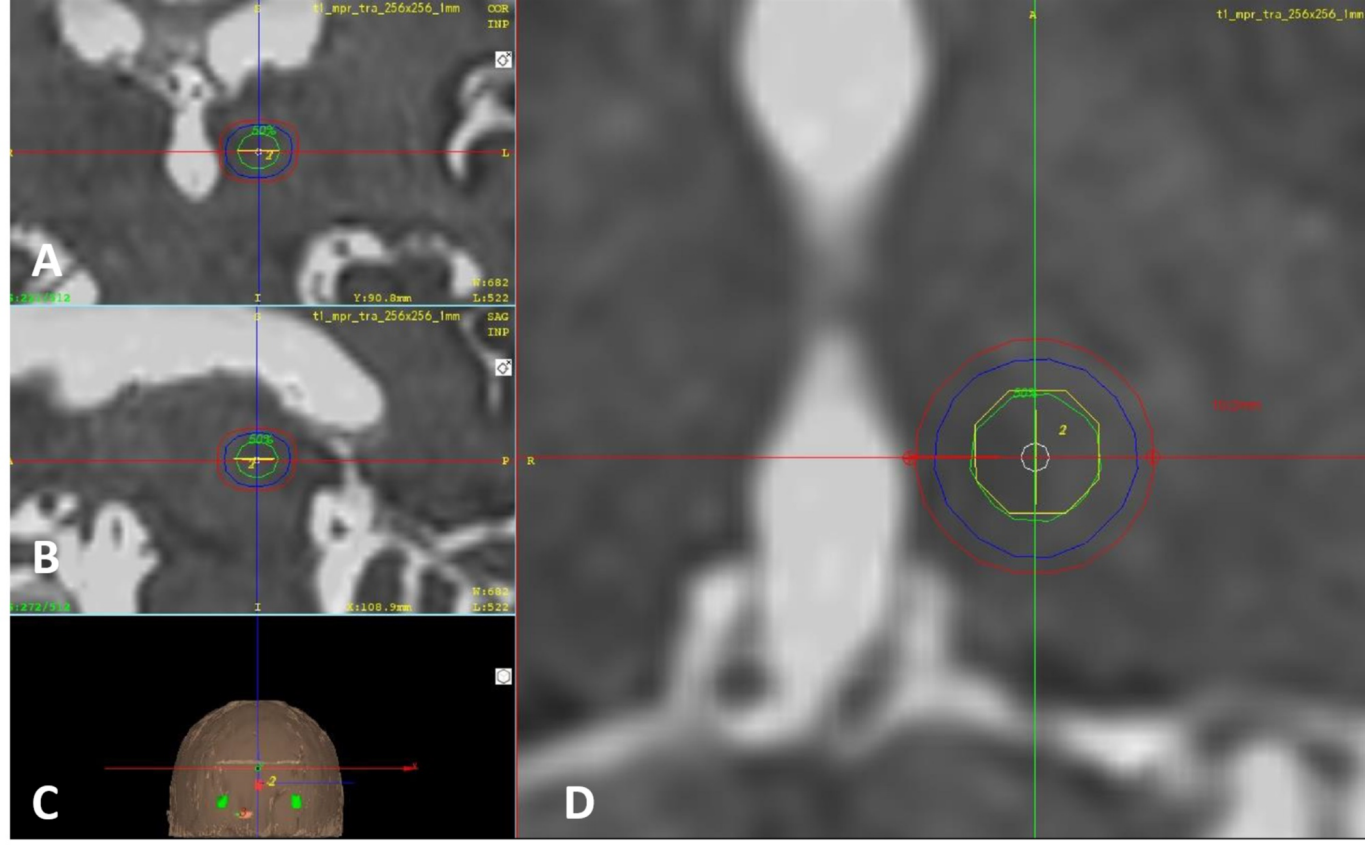
Three-dimensional close-up of the 4-mm shoot (A) Coronal. (B) Sagittal. (C) Three-dimensional reconstruction of the skin. (D) Axial. The green isodose curve represents 50% of the dose (70 Gy), the blue isodose curve in the middle represents dose gradient to 25% of the dose (35 Gy), and the most external red isodose curve represents 24 Gy. The diameter of the area receiving 24 Gy or more of radiation measures 10 mm.

The areas that were irradiated regarding the isocenter and 50% of the Dmax (70 Gy) isodose curve in the present series correspond to the coordinates of the CM-Pf region adjacent and definitely intertwined with the ventroposteromedial (VPM) nucleus that, regardless of the steep dose gradients that characterize Gamma-ray (Knife) use, still cover an area with a diameter of 10 mm from the border of the thalamus to the farthest aspect of the isodose curve corresponding to 24 Gy (Figure 3).

A dose of 24 Gy is known to have a therapeutic effect in epilepsy trials, although this is by no means comparable to the effect of a thalamotomy [22]. The lowest dose to produce a therapeutic or neuromodulation effect by radiosurgery in pain has never been proven, so, relatively high doses of radiation and NER doses above 24 Gy to 70 Gy in the surrounding tissue of the thalamus around the 4-mm shoot cannot be discarded. The VPM is a relay center for the main sensory nucleus and the spinal nucleus of the trigeminal nerve that receives direct afferents from the spinal tract of the nerve. The CM-Pf region also receives afferents from ventroposterolateral and spinothalamic tract as well as the trigeminal lemniscus and finally gives efferent connections to the anterior cingulate cortex. This might explain the affective aspects regarding pain perception and possibly, the NER [16, 23-25]. Further studies are needed using non-ablative technologies such as deep brain stimulation electrodes, micro-registration, or noninvasive low-intensity focused ultrasound to better define the most useful coordinates in this anatomical region of the thalamus and potentially improve clinical results. Bilateral lesions might be able to provide a better result due to the possible ipsilateral tracts to the VPM [24-26]. This series provides further evidence of the importance of this region regarding pain modulation, especially arising from the trigeminal nerve.

Interestingly enough, all the patients in whom the procedure failed reduced their attendance to emergency units for pain management by at least 25%. We noticed that even though the treatment effect was not present at the beginning, family members reported fewer emergency unit visits prior to the feeling of pain relief. Habitual pain medication use was reduced in all but one patient. Due to the characteristics of this investigation and patients’ geographical origins and diverse healthcare systems, we could not adequately quantify these aspects that could potentially be of value.

## Conclusions

This is our first series that describes the clinical results of radiosurgery to the CM-Pf. The results as described seem useful as an alternative technique where most conventional alternatives to control pain have failed or are not viable in trigeminal neuralgia. This procedure seems safe, and three-dimensional atlas imaging provides potentially useful information regarding isodose line distribution in this area that can prompt further studies to refine the best areas to enhance clinical result. Recurrence rates are yet unknown if given enough time after treatment.
